# Mild Malnutrition Contributes the Greatest to the Poor Prognosis in Coronary Artery Disease With Well-Controlled Low-Density Lipoprotein Cholesterol Levels: A 4,863 Chinese Cohort Study

**DOI:** 10.3389/fnut.2021.725537

**Published:** 2021-09-29

**Authors:** Bo Wang, Zhaodong Guo, Jin Liu, Huanqiang Li, Ziling Mai, Feng Lin, Ming Ying, Yaren Yu, Shiqun Chen, Qiang Li, Haozhang Huang, Wen Wei, Yongquan Yang, Shaohong Dong, Yingling Zhou, Jiyan Chen, Ning Tan, Yong Liu

**Affiliations:** ^1^Department of Cardiology, Guangdong Provincial Key Laboratory of Coronary Heart Disease Prevention, Guangdong Cardiovascular Institute, Guangdong Provincial People's Hospital, Guangdong Academy of Medical Sciences, Guangzhou, China; ^2^Department of Dermatology, Shenzhen People's Hospital, The Second Clinical Medical College, Jinan University, The First Affiliated Hospital, Southern University of Science and Technology, Shenzhen, China; ^3^Guangdong Provincial People's Hospital, School of Medicine, South China University of Technology, Guangzhou, China; ^4^Department of Cardiology, The First People's Hospital of Foshan, Foshan, China; ^5^Guangdong Provincial People's Hospital, The Second School of Clinical Medicine, Southern Medical University, Guangzhou, China; ^6^Department of Endocrinology, Longyan First Hospital Affiliated With Fujian Medical University, Longyan, China

**Keywords:** mild nutrition, low-density lipoprotein cholesterol, coronary artery disease, long-term all-cause mortality, risk factor, population attributable risk

## Abstract

**Background:** Previous studies reported that patients with coronary artery disease (CAD) and well-controlled baseline LDL-C (<1.8 mmol/L) still had higher long-term all-cause mortality. However, no study has been conducted to explore the independent risk factors for long-term mortality. In addition, there also was no study evaluating the population attributable risk (PAR) of independent risk factors in combination with their prevalence and relative risk. Therefore, we aimed to identify the independent risk factors and estimate their PAR in patients with CAD and well-controlled baseline LDL-C (<1.8 mmol/L).

**Methods:** We analyzed 4,863 consecutive CAD patients with well-controlled baseline LDL-C admitted to Guangdong Provincial People's Hospital in China from January 2007 to December 2018. Independent risk factors for long-term all-cause death were evaluated through stepwise approach and multivariable Cox regression analysis. PAR of independent risk factors was calculated with their hazard ratio and prevalence among our cohort.

**Results:** The overall mortality was 16.00% (n = 778) over a median follow-up period of 5.93 years. Independent risk factors for all-cause death included malnutrition, age ≥75 years, congestive heart failure (CHF), chronic kidney disease (CKD) and atrial fibrillation. Among these risk factors of interest, the hazard ratio (HR) of severe malnutrition was the highest (HR 2.82, 95% CI: 1.86–4.26), and the PAR of mild malnutrition was the highest (19.49%, 95% CI: 0.65–36.01%).

**Conclusion:** Malnutrition, age ≥75 years, CHF, CKD and atrial fibrillation were independent predictors for long-term all-cause mortality in CAD patients with well-controlled LDL-C levels. Considering prevalence of these risk factors, more attention should be paid to the occurrence of mild malnutrition for these patients.

**Clinical Trial Registration:** ClinicalTrials.gov, identifier: NCT04407936.

## Introduction

It has been established that serum low-density lipoprotein cholesterol (LDL-C) is a key risk factor for coronary artery disease (CAD) (1–3). Guidelines also recommended LDL-C as a target for the primary and secondary prevention of CAD (1). However, LDL-C levels are not always elevated in patients with CAD (4, 5). Previous study showed CAD patients with well-controlled baseline LDL-C (<1.8 mmol/L) had higher long-term all-cause mortality than those with poorly controlled baseline LDL-C levels (≥1.8 mmol/L) (6). Currently, there is not enough information on the management of this population improving prognosis for clinicians. It is necessary to identify the independent predictors of all-cause death.

In clinical practice, prevalence of risk factors should also be taken into account. The population attributable risk (PAR) combines the relative risk of a risk factor with its prevalence in a given population to determine the proportion of outcomes associated with (or attributable to) that factor (7). It can evaluate the prevalence and risk degree of risk factors comprehensively. Therefore, this study aims to determine the independent predictors of mortality for CAD patients with baseline well-controlled LDL-C levels and estimate their PAR among a large CAD cohort.

## Method

### Study Design and Participants

**Figure 1:**
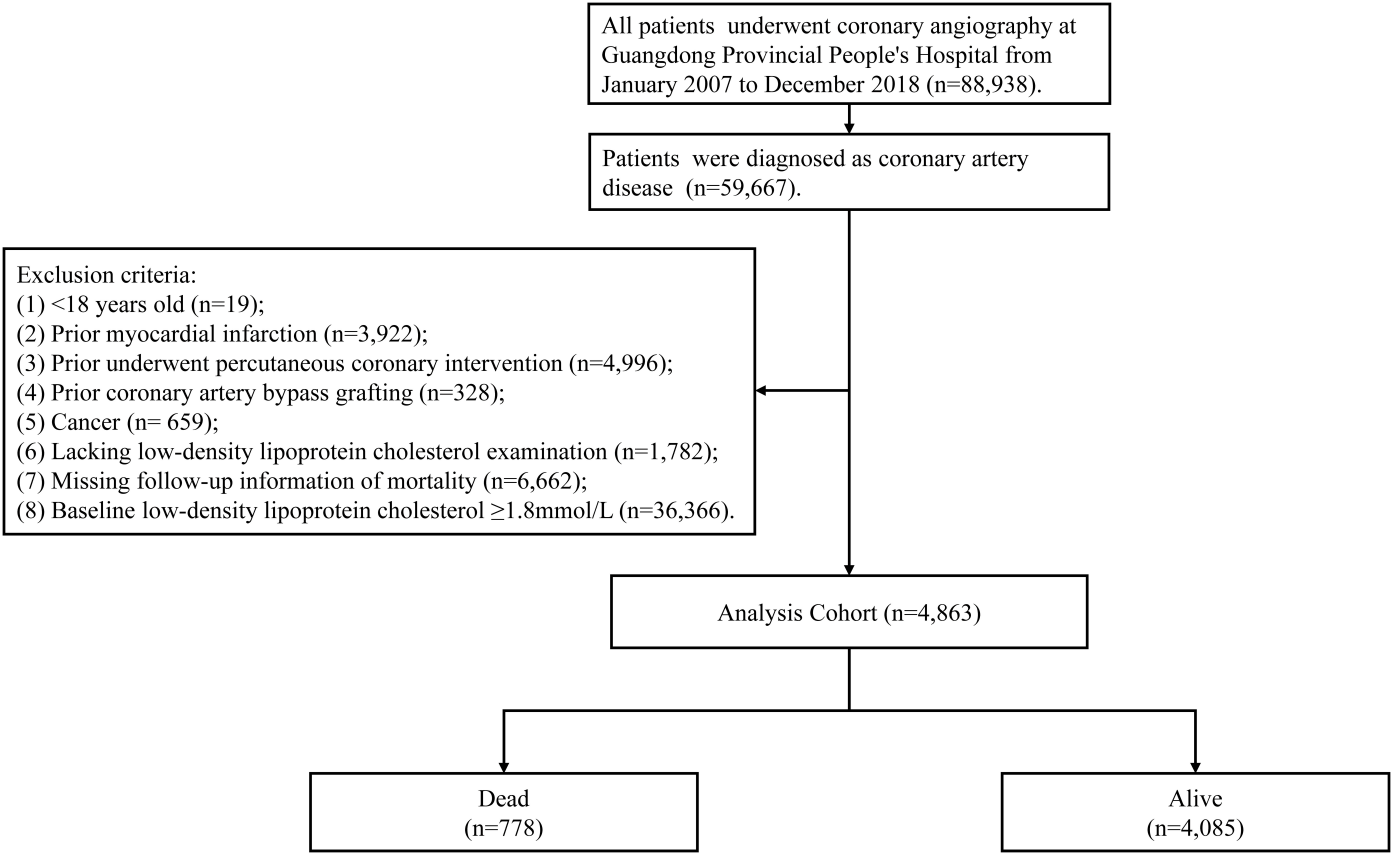
Study flow chart.

The retrospective observational study was conducted in Guangdong Provincial People's Hospital, China (Clinicaltrials.gov NCT04407936) from January 2007 to December 2018. During this period, there were 88,938 patients underwent coronary angiography (CAG) and 59,667 patients were diagnosed as CAD. We excluded patients <18 years old (*n* = 19), prior myocardial infarction (*n* = 3,922), prior undergoing PCI (*n* = 4,996), prior undergoing coronary artery bypass grafting (*n* = 328), cancer (*n* = 659), lacking LDL-C examination (*n* = 1,782), missing follow-up information of mortality (*n* = 6,662) and LDL-C level ≥1.8 mmol/L(*n* = 36,366). A total of 4,863 patients were eventually included in the study (Figure 1). The study protocol was approved by the Ethics Committee of Guangdong Provincial People's Hospital (No. GDREC2019555H[R1]) and was conducted in accordance with the Declaration of Helsinki.

### Procedures

The clinical information for each patient in cohort was extracted from the electronic Clinical Management System of the Guangdong Provincial People's Hospital. Baseline data were collected on demographic characteristics, coexisting conditions, laboratory tests, nutritional status and medication at discharge. In all patients, blood samples except lipid profiles were collected at admission or before coronary angiography (CAG) and percutaneous coronary intervention (PCI). The lipid profiles were measured by fasting blood samples which were collected between 06:00 and 08:00 in the morning. For patients admitted between 0:00 and 06:00, blood samples were collected between 06:00 and 08:00 in the morning of the same day. For the remaining patients, blood samples were collected between 06:00 and 08:00 on the next day after admission. CAG/PCI was performed in accordance with the standard clinical practice guidelines (8–10). Senior cardiologists were responsible for the data quality control and periodical data verification. Follow-up data were monitored and recorded by trained nurses through outpatient interviews and telephone follow-up. The follow-up information of patients lost to follow-up was retrieved from Guangdong Public Security System.

### Clinical Outcome and Definition

The primary endpoint was long-term all-cause mortality. CAD was confirmed by CAG and discriminated according to the 10th Revision Codes of the International Classification of Diseases. Comorbidities included acute myocardial infarction (AMI), diabetes mellitus, hypertension, chronic kidney disease (CKD), atrial fibrillation, chronic obstructive pulmonary disease (COPD), congestive heart failure (CHF) and anemia. CHF was defined as New York Heart Association (NYHA) class >2 or Killip class >1 (11). CKD was recognized as estimated glomerular filtration rate (eGFR) ≤ 60 mL/min/1.73 m^2^ and the eGFR was determined by the Modification of Diet in Renal Disease (MDRD) formula (12–14). Anemia was defined as hematocrit <39% for men and hematocrit <36% for women, according to the World Health Organization criteria (15). Nutritional status was assessed by the Controlling Nutritional Status (CONUT) score, a screening tool for the nutritional status of hospitalized patients (16). It was calculated by serum albumin, cholesterol, and total lymphocyte count. Different scores represent different nutritional status (0–1 is normal; 2–4 is mild malnutrition; 5–8 is moderate malnutrition; 9–12 is severe malnutrition).

### Statistical Analysis

Patients were divided into 2 groups: those who died or survived during the follow-up period. Data are reported as mean (SD), median (interquartile range [IQR]), or numbers and percentages. Differences of continuous variables with normal or non-normal distributions between two groups were evaluated using independent sample student *t*-test or Wilcoxon rank sum test. Categorical variables were tested using Pearson chi-square test.

Risk factors which were significantly different in the two groups and with missing value <15% were included in the univariable Cox regression analysis to preliminarily determine the association with long-term all-cause mortality. When interaction or collinearity existed between variables, the modifiable or categorical one was preferred to facilitate clinical application. Correlations between LDL-C and nutritional status and its components were assessed by the Spearman correlation test. Then variables associated with all-cause mortality with p < 0.05 in the univariate analysis were enrolled in a Cox regression analysis with backward stepwise selection to determine the independent predictors of all-cause mortality in CAD patients with well-controlled LDL-C levels. Multivariable Cox regression model including all the remaining risk factors was then fitted. The results were reported as the adjusted hazard ratio (HR) and associated 95% confidence intervals (CI). Furthermore, to comprehensively consider the prevalence and HR of risk factors, the study calculated the population attributable risk (PAR). PAR was calculated using the equation PAR = P (HR-1)/ [1 + P (HR-1)], where P is the prevalence of eventually determined risk factor in our cohort. The standard error of PAR was calculated using the delta method (17). All data analysis was performed by R software, version 3.6.3 (R Foundation for Statistical Computing). The results of all analyses with *p* < 0.05 were considered statistically significant.

## Result

### Baseline Characteristic

Continuing with previous studies (6), this study included 4,863 CAD patients with well-controlled baseline LDL-C level (<1.8 mmol/L). Among all recruited patients, the long-term all-cause mortality rate was 16.00% (*n* = 778) during a median follow-up of 5.93 (IQR 4.08–8.40) years. Table 1 detailed the patient's clinical characteristics. Compared with long-term survivors, those who died during the follow-up period were more likely to be older and had higher prevalence of AMI, CHF, CKD, atrial fibrillation and anemia. In addition, the rates of moderate malnutrition and severe malnutrition were also higher.

**Table 1:** Baseline characteristics.

Characteristic^*^	**Overall**	**All-cause mortality**		***P*** **-value**
	**(*N* = 4,863)**	**Dead (*N* = 778)**	**Alive (*N* = 4,085)**	
**Demographic characteristics**				
Age, year	64.38 (10.81)	67.25 (11.35)	63.83 (10.62)	<0.001
Age ≥75 years, *n* (%)	929 (19.10)	237 (30.46)	692 (16.94)	<0.001
Male, *n* (%)	3,725 (76.60)	589 (75.71)	3,136 (76.77)	0.55
**Coexisting conditions**				
PCI, *n* (%)	3,433 (70.59)	538 (69.15)	2,895 (70.87)	0.36
AMI, *n* (%)	805 (16.55)	169 (21.72)	636 (15.57)	<0.001
CHF, *n* (%)	409 (8.42)	117 (15.04)	292 (7.16)	<0.001
Hypertension, *n* (%)	3,020 (62.10)	489 (62.85)	2,531 (61.96)	0.67
Diabetes mellitus, *n* (%)	1,623 (33.37)	271 (34.83)	1,352 (33.10)	0.37
CKD, *n* (%)	1,154 (24.84)	295 (39.60)	859 (22.02)	<0.001
Atrial fibrillation, *n* (%)	133 (2.73)	31 (3.98)	102 (2.50)	0.03
COPD, *n* (%)	54 (1.11)	12 (1.54)	42 (1.03)	0.29
Stroke, *n* (%)	347 (7.14)	62 (7.97)	285 (6.98)	0.36
Anemia, *n* (%)	2079 (43.98)	404 (54.74)	1,675 (41.99)	<0.001
**Nutritional status**				
Normal, *n* (%)	446 (9.70)	51 (7.12)	395 (10.17)	<0.001
Mild malnutrition, *n* (%)	3,010 (65.43)	393 (54.89)	2,617 (67.38)	<0.001
Moderate malnutrition, *n* (%)	1,020 (22.17)	220 (30.73)	800 (20.60)	<0.001
Severe malnutrition, *n* (%)	124 (2.70)	52 (7.26)	72 (1.85)	<0.001
**Laboratory examination**				
HCT	0.38 (0.05)	0.37 (0.06)	0.39 (0.05)	<0.001
Lymphocyte, 10^9^/L	1.83 (0.69)	1.68 (0.64)	1.85 (0.69)	<0.001
Neutrophil, 10^9^/L	5.05 (2.72)	5.79 (3.64)	4.92 (2.49)	<0.001
Total cholesterol, mmol/L	3.24 (0.91)	3.22 (0.78)	3.24 (0.93)	0.53
HDL-C, mmol/L	0.93 (0.30)	0.92 (0.31)	0.93 (0.30)	0.35
LDL-C, mmol/L	1.47 (0.26)	1.46 (0.27)	1.48 (0.26)	0.13
Triglyceride, mmol/L	1.68 (1.96)	1.61 (1.64)	1.70 (2.02)	0.28
Non-HDL-C, mmol/L	2.31 (0.85)	2.30 (0.70)	2.31 (0.88)	0.74
ALB, g/L	35.74 (4.44)	34.00 (4.85)	36.06 (4.28)	<0.001
**Medicine**				
RASi, *n* (%)	2,199 (46.46)	343 (48.65)	1,856 (46.08)	0.22
β-blocker, *n* (%)	3,776 (79.78)	545 (77.30)	3,231 (80.21)	0.09
Statins, *n* (%)	4,396 (92.88)	638 (90.50)	3,758 (93.30)	0.01

* *Data are presented as the mean value (standard deviation), median [interquartile range] or number of participants (percentage). PCI, percutaneous coronary intervention; AMI, acute myocardial infarction; CHF, congestive heart failure; CKD, chronic kidney disease; COPD, chronic obstructive pulmonary disease; HDL-C, high-density lipoprotein cholesterol; LDL-C, low-density lipoprotein cholesterol; RASi, renin angiotensin system inhibitor*.

### Correlations Between LDL-C and Nutritional Status and Its Components

**Figure 2:**
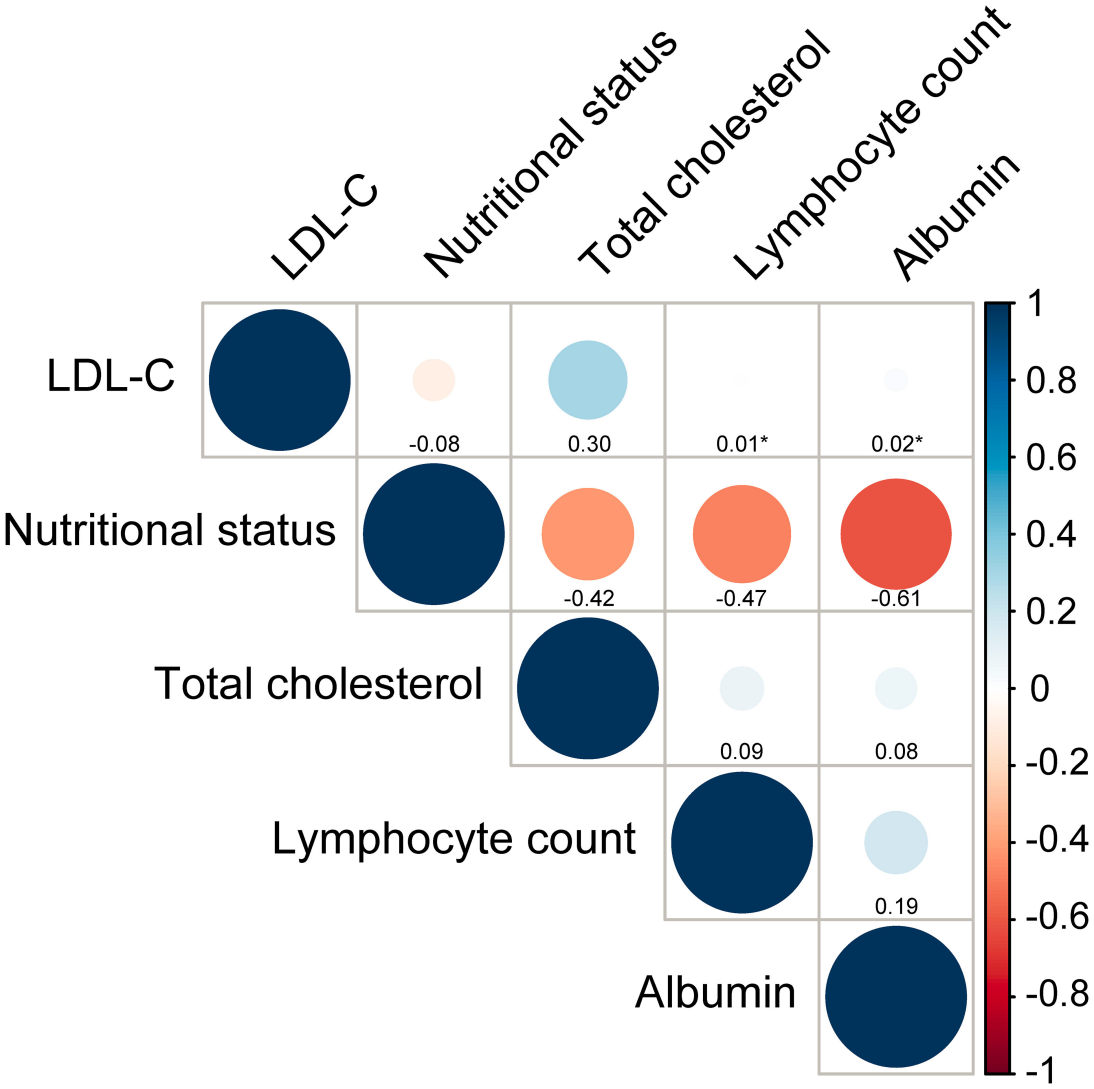
Correlations between LDL-C and nutritional status and its components. Nutritional status is assessed by Controlling Nutritional Status (CONUT) score. Total cholesterol, lymphocyte count and albumin are components of CONUT score. *p-value for spearman correlation >0.05.

Figure 2 showed all correlations between LDL-C and nutritional status evaluated by CONUT score and components of CONUT score. LDL-C did not show a significant correlation with nutritional status (r = −0.08, *p* < 0.001). As for the correlations between LDL-C and components of CONUT score, there was no strong correlation between LDL-C and total cholesterol (r = 0.30, *p* < 0.001). Additionally, LDL-C was not correlated with lymphocyte count and albumin (*p* = 0.65 and 0.14).

### Risk Factors for All-Cause Mortality

The multivariate Cox regression analysis with backward stepwise identified that age ≥75 years (HR 1.61, 95% CI: 1.37–1.91), CHF (HR 1.84, 95% CI: 1.48–2.28), CKD (HR 1.71, 95% CI: 1.46–2.01), atrial fibrillation (HR 1.93, 95% CI: 1.34–2.79), mild malnutrition (HR 1.37, 95% CI: 1.01–1.86), moderate malnutrition (HR 1.79, 95% CI: 1.30–2.47) and severe malnutrition (HR 2.82, 95% CI: 1.86–4.26) were independently associated with long-term all-cause mortality (Table 2). The results of Cox model with additional adjustment for AMI were showed in Supplementary Table 1, which were similar as the results in above multivariate model.

**Table 2:** Univariate regression analysis and multivariate analysis with backward stepwise for the association between all-cause death and clinical findings during follow-up period.

	**Univariate analysis**			**Multivariate analysis**		
	**HR**	**95% CI**	***P*** **-value**	**HR**	**95% CI**	***P*** **-value**
Age ≥75 years	2.04	(1.75–2.38)	<0.001	1.61	(1.37–1.91)	<0.001
Male	0.95	(0.81–1.12)	0.56			
PCI	0.90	(0.78–1.05)	0.20			
AMI	1.32	(1.11–1.56)	0.002			
CHF	2.50	(2.05–3.04)	<0.001	1.84	(1.48–2.28)	<0.001
Hypertension	1.11	(0.96–1.28)	0.17			
Diabetes mellitus	1.12	(0.97–1.3)	0.13			
CKD	2.09	(1.8–2.42)	<0.001	1.71	(1.46–2.01)	<0.001
Atrial fibrillation	2.50	(1.74–3.59)	<0.001	1.93	(1.34–2.79)	<0.001
COPD	1.38	(0.78–2.43)	0.27			
Stroke	1.32	(1.02–1.71)	0.04			
Anemia	1.57	(1.35–1.81)	<0.001			
Mild malnutrition vs normal	1.43	(1.06–1.91)	0.02	1.37	(1.01–1.86)	0.04
Moderate malnutrition vs. normal	2.30	(1.70–3.12)	<0.001	1.79	(1.30–2.47)	<0.001
Severe malnutrition Vs. normal	5.20	(3.53–7.65)	<0.001	2.82	(1.86–4.26)	<0.001

*HR, hazard ratio; CI, confidence interval; PCI, percutaneous coronary intervention; AMI, acute myocardial infarction; CHF, congestive heart failure; CKD, chronic kidney disease; COPD, chronic obstructive pulmonary disease*.

### PAR of Risk Factors for All-Cause Mortality

**Figure 3:**
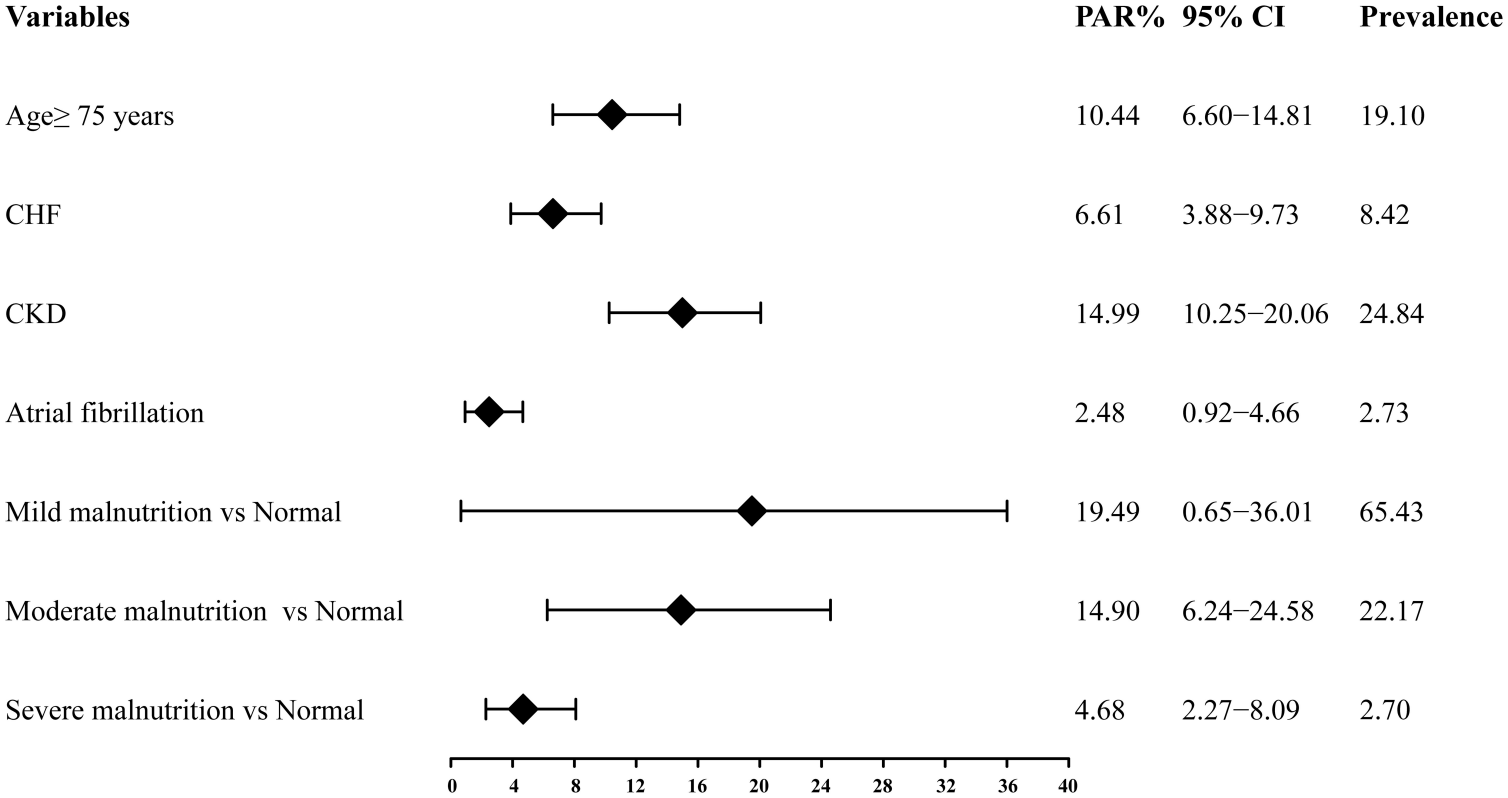
Population attributable risk of the independent risk factors of long-term all-cause mortality. PAR, population attributable risk; CI, confidence interval; CHF, congestive heart failure; CKD, chronic kidney disease.

Among these risk factors of interest of all-cause death, the prevalence was highest for mild malnutrition (65.43%), followed by CKD (24.84%), moderate malnutrition (22.17%), age ≥75 years (19.10%), CHF (8.42%), atrial fibrillation (2.73%), and the lowest was 2.70% for severe malnutrition. The PAR was highest for mild malnutrition (19.49, 95% CI: 0.65–36.01%), followed by CKD (14.99, 95% CI: 10.25–20.06%), moderate malnutrition (14.90, 95% CI: 6.24–24.58%), age ≥75 years (10.44, 95% CI: 6.60–14.81%), CHF (6.61, 95% CI: 3.88–9.73%), severe malnutrition (4.68, 95% CI: 2.27-8.09%) and atrial fibrillation (2.48, 95% CI: 0.92–4.66%) (Figure 3). The PAR based on Cox model with additional adjustment for AMI were showed in Supplementary Table 1, which were similar as the above results.

## Discussion

To our acknowledge, it is the first study to explore the independent risk factors of long-term all-cause mortality in CAD patients with well-controlled LDL-C levels. Furthermore, this study estimated the proportion of all-cause mortality attributed to four risk factors by calculating PAR. The relative risk was highest for severe malnutrition. Considering the prevalence of identified risk factors, the highest PAR was found for mild malnutrition, followed by CKD, moderate malnutrition, age ≥75 years, CHF, severe malnutrition and atrial fibrillation.

Among these identified risk factors, malnutrition showed a significant association with poor prognosis. Previous studies reported many nutrition assessment tools, categorized as subjective nutritional screening assessment tools and objective nutrition screening tools (18). Subjective nutritional screening assessment tools such as Subjective Global Assessment (SGA) (19), Mini Nutritional Assessment (MNA) (20), Nutritional Risk Index (NRI) (21), etc. require anthropometric measurements, indication of disease severity, clinical assessment, or an extensive questionnaire addressing several aspects of nutritional intake. Although these tools can evaluate the nutrition status comprehensively, they are complex and time-consuming, which may not be convenient in clinical practice. Objective nutrition screening tools evaluate nutritional status by height, weight and/ or blood parameters which are readily available from routine clinical laboratory examination. These tools including CONUT score (16), Prognostic Nutritional Index (PNI) (22), Geriatric Nutritional Risk Index (GNRI) (23), etc. are convenient in clinical practice. Previous studies demonstrated that the CONUT score has been demonstrated as a suitable tool for evaluating the nutritional status among CAD patients (24). In our study, we useed the CONUT score as the screening tool to assess patients' nutritional status. Compared with normal nutritional status, each type of malnutrition increased the risk of all-cause mortality. Risks increased by 37%, 79% and 182% in mild, moderate and severe malnutrition. Severe malnutrition had the highest risk degree among all of the determined independent predictors. A retrospective observational study conducted by (24) also drew the same conclusion (24). Their study enrolled 5,062 patients with acute coronary syndrome and found that all three levels of malnutrition increased the risk of all-cause mortality after adjusting for confounding factors. Mild, moderate and severe malnutrition increases the risk of all-cause death by 36, 102, and 265%, respectively. In another observational cohort study, included 1,987 patients with stable CAD who underwent elective PCI (25). This study divided patients into two groups according to their CONUT score. One group was nutritionally normal and the other was malnourished which included mild, moderate and severe malnutrition. Findings of this study demonstrated that malnutrition increased the risk of a composite outcome of all-cause death and non-fatal MI by 64%. Although the relative risk of severe malnutrition is the highest, it only occurs in 2.70% of enrolled patients. After considering the prevalence of identified risk factors in the current study, our results indicated that mild malnutrition was associated with 19.49% of the occurrence of long-term all-cause mortality, which was the highest. Mild malnutrition is also the most common type of malnutrition. In the above two studies, mild malnutrition accounted for the largest proportion of malnutrition. Our results show the same phenomenon. Among all the patients, malnutrition accounted for 90.30%, and in the malnutrition, mild malnutrition accounted for 72.46%. Previous studies reported that concentration of LDL-C and total cholesterol decreased during the acute phase of AMI. acute phase of AMI. A prospective observational study involving 1,275 AMI patients, illustrated that total cholesterol and LDL-C decreased 7% and 10% after admission compared to baseline (26). Similar results were also be found in other three studies (27–29). This may lead to lower total cholesterol level in AMI patients, leading to worse nutritional status. However, our study found no effect on results after extra adjustment for AMI.

Nutritional status assessed by the CONUT score which consists of three laboratory markers: albumin, cholesterol and lymphocyte count, also reflects protein and lipid metabolism as well as immune function measured from blood tests (30). Lower albumin levels reflect increased production of catabolic cytokines, muscle catabolism, and appetite suppression, which are associated chronic inflammatory diseases (31). Cholesterol homeostasis occurs as part of innate immune response and its disruption may augment inflammatory responses (32). There are also evidences that cholesterol concentration is associated with the regulation of immune cell function which can improve antitumour activity and activate immune signaling (33–35). Lymphocyte counts reflect the host immunity and have been studied with respect to their association with nutritional status (36). A low lymphocyte percentage was shown to be independently and significantly associated with worse prognosis (37). Failure to recognize or to anticipate the development of malnutrition can increase morbidity and mortality.

Our study also showed that CKD was an independent risk factor of long-term prognosis, which was consistent with previous studies (38, 39). The results indicated that CKD had the second highest PAR for all-cause mortality and was associated with 14.99% of the death cases. Indeed, CKD is one of the major causes of long-term mortality among patients with CAD. Even after adjustment for traditional risk factors of CAD, including age, dyslipidemia, diabetes, and hypertension, the risk of long-term mortality linearly increases with deteriorating kidney function (40, 41). Previous researches have illustrated that as glomerular filtration rate (GFR) declines below 60 to 75 ml/min/1.73 m^2^, the risk of developing CAD increases linearly (12, 41), especially in those patients with CKD stages G3a to G4 (15-60 ml/min/1.73 m^2^), which have about double and triple the risk of cardiovascular death, respectively, compared with those in the non-CKD setting. With the decline of GFR, the prevalence of clinical symptoms of CAD increases, along with the prevalence of multivessel coronary artery disease, severe coronary calcification, longer atherosclerotic lesions with greater luminal encroachment, microangiopathy, left ventricular hypertrophy (LVH), and higher plaque burden. Cardiovascular malformation in patients with CKD is associated with identified (e.g., diabetes, dyslipidemia, and hypertension), potential CKD-related risk factors of CAD (e.g., CKD-mineral and bone disorder, anemia, inflammation, and oxidative stress) and the dialysis-associated factors (type, frequency, and pattern of continuous renal replacement therapy). With the continuous deterioration of renal function, the calcification of vessels also increases and is associated with mortality in advanced kidney disease (AKD); both severe calcification of the subintima and media of large vessels are associated with adverse long-term prognosis. Various studies suggest that there are significant opportunities to improve treatment of identified risk factors, and KDIGO guidelines for lipid management in CKD provide specific treatment recommendations (42). Nevertheless, there are several reasons why treatment of established risk factors of CVD is lacking, including weak evidence of effectiveness or extrapolation of evidence from the non-CKD setting. Continued work is required to better understand the interaction between CAD, CKD, and prognosis.

Based on the findings of the current study, CAD patients with well-controlled baseline LDL-C levels still have higher all-cause mortality. According to determined risk factors and their prevalence, malnutrition contributes greatly to the poor prognosis in this population. Among different degrees of malnutrition, mild malnutrition is the most common and is often overlooked by clinicians. However, mild malnutrition contributes the greatest to poor prognosis, which requires clinician's attention and timely intervention. Furthermore, mild malnutrition may be a target in the long-term management of such patients, and new prospective study involving prognosis management in these patients should be conducted to evaluate the appropriate management strategy.

## Limitation

There were some limitations to this analysis. First, this study was a retrospective single-center study, so the prevalence of the risk factors may not be representative enough. However, Guangdong Provincial People's Hospital is the largest cardiology center in South China, and PAR can only be calculated based on observational data. Second, data of included patients were limited, without information about body weight, BMI, waist circumference or adiposity, which might help to assess nutritional status comprehensively. However, we chose the CONUT score based on three laboratory indicators as a nutritional status assessment tool. This may also objectively evaluate the nutritional status of the patient. Third, LDL-C level and total concentration decrease at the acute phase of AMI. Thus, LDL-C value and total cholesterol value before admission or later after admission may be more suitable for AMI patients. However, our study lacks such data. Finally, since this was an observational study, we can only suggest that modifying these risk factors may lead to a lower risk of long-term all-cause mortality rather than prove it. The value our results add is in providing these cost-effective targets for further intervention trials.

## Conclusion

Malnutrition, age ≥75 years, CHF, CKD and atrial fibrillation were independent predictors for long-term all-cause mortality in CAD patients with well-controlled LDL-C levels. Severe malnutrition had the highest HR. Considering the prevalence of these risk factors, mild malnutrition contributed the greatest to the long-term all-cause mortality. More attention should be paid to mild malnutrition among patients with CAD and well-controlled LDL-C level (<1.8 mmol/L).

## Data Availability Statement

The datasets analyzed during the current study will be available from the corresponding author on a reasonable request when the study is finished. Requests to access the datasets should be directed to the corresponding author, Yong Liu, liuyong@gdph.org.cn.

## Ethics Statement

The studies involving human participants were reviewed and approved by Research Ethics Committee of Guangdong Provincial People's Hospital, Guangdong Academy of Medical Sciences (No. GDREC2019555H[R1]). Written informed consent for participation was not required for this study in accordance with the national legislation and the institutional requirements.

## Author Contributions

YL and SC had full access to data and took responsibility for the data analyses integrity and accuracy. YL and BW: concept and design. YoY: data management. BW, ZG, FL, HL, ZM, MY, YaY, QL, HH, and WW: drafting of the manuscript. JC, YZ, and SD: critical revision. NT and YL: final approval to publish. All authors contributed to acquisition, analyses and interpretation of data, and read and approved the final manuscript.

## Funding

This study was supported by the National Key Research and Development Program of China (Grant no. 2016YFC1301202), the Beijing Lisheng Cardiovascular Health Foundation (LHJJ20141751), Guangdong Provincial Science and Technology Plan Project (2017B030314041) and Guangdong Provincial People's Hospital Dengfeng Project Fund (DFJH201919 and DFJH2020026).

## Conflict of Interest

The authors declare that the research was conducted in the absence of any commercial or financial relationships that could be construed as a potential conflict of interest.

## Publisher's Note

All claims expressed in this article are solely those of the authors and do not necessarily represent those of their affiliated organizations, or those of the publisher, the editors and the reviewers. Any product that may be evaluated in this article, or claim that may be made by its manufacturer, is not guaranteed or endorsed by the publisher.

## Abbreviations

LDL-C: low-density lipoprotein cholesterol
CAD: coronary artery disease
CAG: coronary angiography
PCI: percutaneous coronary intervention
AMI: acute myocardial infarction
CHF: congestive heart failure
CKD: chronic kidney disease
COPD: chronic obstructive pulmonary disease
HR: hazard ratio
CI: confidence interval.
