# Supplementary material for: Mild Malnutrition Contributes the Greatest to the Poor Prognosis in Coronary Artery Disease With Well-Controlled Low-Density Lipoprotein Cholesterol Levels: A 4,863 Chinese Cohort Study
Source: Front Nutr. 2021 Sep 29;8:725537. doi: 10.3389/fnut.2021.725537 (PMC8511711; doi:10.3389/fnut.2021.725537)
Supplement: Supplementary file 1 [file Table_1.DOCX]

**Supplementary Table 1.** Multivariate Cox regression and PAR for risk factors of long-term all-cause mortality (additional adjustment for AMI)

|  | HR | 95% CI | *P* Value | PAR% | 95% CI | Prevalence |
| --- | --- | --- | --- | --- | --- | --- |
| Age ≥75 years | 1.62 | 1.37-1.91 | <0.001 | 10.59 | 6.60-14.81 | 19.10% |
| CHF | 1.81 | 1.44-2.28 | <0.001 | 6.38 | 3.57-9.73 | 8.42% |
| CKD | 1.72 | 1.46-2.01 | <0.001 | 15.17 | 10.25-20.06 | 24.84% |
| Atrial fibrillation | 1.94 | 1.35-2.81 | <0.001 | 2.50 | 0.95-4.71 | 2.73% |
| Mild malnutrition  vs. normal | 1.37 | 1.01-1.86 | 0.04 | 19.49 | 0.65-36.01 | 65.43% |
| Moderate malnutrition  vs. normal | 1.77 | 1.28-2.45 | 0.001 | 14.58 | 5.84-24.33 | 22.17% |
| Severe malnutrition  vs. normal | 2.79 | 1.84-4.23 | <0.001 | 4.61 | 2.22-8.02 | 2.70% |
| AMI | 1.04 | 0.85-1.27 | 0.71 | 0.66 | -2.55-4.28 | 16.55% |

Abbreviations: PAR, population attributable risk; HR, hazard ratio; CI, confidence interval; CHF, congestive heart failure; CKD, chronic kidney disease; AMI, acute myocardial infarction.

.
